# A randomised pilot study evaluating music therapy and virtual reality mindfulness sessions for reducing anxiety and stress in patients undergoing first-time elective cardiac surgery

**DOI:** 10.1177/17504589251370291

**Published:** 2025-10-04

**Authors:** Bhuvaneswari Krishnamoorthy, Moslem Abdelghafar, Rick Air, Joshua Halyckyj-Smith, Angelo Citarella, Edward McLaughlin, Heather Iles-Smith, Rebecca Elliott, James Barnard, Azita Rajai, Rajamiyer Venkateswaran

**Affiliations:** 1 Directorate of Nursing and Midwifery and School of Health and Society, University of Salford, Salford, UK; 2 Department of Cardiothoracic Surgery, Manchester Foundation Trust, Wythenshawe Hospital, Manchester, UK; 3 Faculty of Health, Department of Cardiovascular Sciences, Biology and Medicine, University of Manchester, Manchester, UK; 4 Faculty of Biology, Division of Psychology and Mental Health, Medicine and Health, University of Manchester, Manchester, UK; 5 Manchester University NHS Foundation Trust, Manchester Academic Health Science Centre, Manchester, UK; 6 Centre for Biostatistics, Division of Population Health, University of Manchester, Manchester, UK

**Keywords:** Virtual reality, Cardiac surgery, Mindfulness, Wellbeing, Anxiety, Stress, Coronary artery bypass surgery, Music intervention

## Abstract

Cardiac surgery patients may experience stress and anxiety, negatively impacting recovery. Pharmacological treatments are frequently used but may result in side effects. We aimed to assess the effectiveness of immersive virtual reality mindfulness and music therapy in reducing preoperative and postoperative anxiety. Between March and July 2024, 36 participants were randomised to 15 min of either virtual reality (*n* = 19) or music therapy (*n* = 17) preoperatively and on postoperative day 3. Primary endpoints were anxiety levels and salivary cortisol, assessed before and after intervention using the paired Wilcoxon test. Median age was 66 (interquartile range = 58–73); 64% were male. Both music therapy and virtual reality groups showed significant preoperative Spielberger State-Trait Anxiety Inventory score reductions after intervention: music therapy from 40 (30–48) to 23 (21–24) and virtual reality from 40 (31–54) to 23 (20–35) (both *p* < 0.001); there was no significant difference between groups (*p* = 0.7). Median virtual reality immersion and absorption scores were 90/100 (interquartile range = 80–90; 80–94). Cortisol levels did not significantly change. Postoperatively, 21 patients participated (virtual reality *n* = 11, music therapy *n* = 8), showing reduced anxiety: music therapy from 31 (26–32) to 23 (20–27), virtual reality from 33 (23–41) to 24 (22–30). Results show feasibility, tolerability, and preliminary evidence of anxiety reduction, supporting further research into music therapy and virtual reality for cardiac surgery patients. The trial was registered with the International Standard Randomised Controlled Trial Number Registry (ISRCTN51014051) (https://doi.org/10.1186/ISRCTN51014051).

## Introduction

Patients undergoing surgery frequently experience significant levels of stress and anxiety, with the incidence and severity of these symptoms varying widely across different medical specialties (Yu et al 2022). Cardiac surgery patients, in particular, face notably high levels of stress due to the perceived gravity of the surgery and apprehension of the potential complications (Fathi et al 2014, Prado-Olivares & Chover-Sierra 2019). Studies have found that moderate preoperative anxiety is present in up to 10%–20% and severe anxiety up to 75%–80% of patients undergoing cardiac surgery (Mudgalkar et al 2022). Preoperative anxiety can negatively impact surgical outcomes by affecting mental wellbeing and contributing to physiological responses such as hypertension, tachycardia, and increased intraoperative bleeding, among other complications (Guo 2015).

Traditionally, pharmacological interventions have been employed to manage preoperative anxiety. Sedatives and anxiolytics are commonly used, but they come with drawbacks such as transient mucosal irritation, postoperative nausea and vomiting, emergence delirium, and extended hospital stay (Fathi et al 2014, Lee & Sung 2020). Consequently, there has been a growing interest in non-pharmacological interventions, which offer alternative or complementary strategies to traditional pharmacologic methods (Manyande et al 2015).

Distraction approaches such as videos, multifaceted programmes, interactive games, and virtual reality (VR) have shown promise, particularly in paediatric populations (Chow et al 2016, Eijlers et al 2019, Tas et al 2022). Although the evidence in adult populations is less conclusive, several studies suggest potential benefits. For instance, a Randomised Controlled Trial (RCT) involving adult patients using VR before surgery reported a significant reduction in anxiety levels, highlighting VR’s potential as an effective distraction tool (Chiu et al 2023).

This study aimed to assess immersive head-mounted VR-guided mindfulness compared to music therapy (MT) in adult patients undergoing elective/urgent in-patient cardiac surgery to understand the impact on patient anxiety and stress.

## Methods

**Figure 1:**
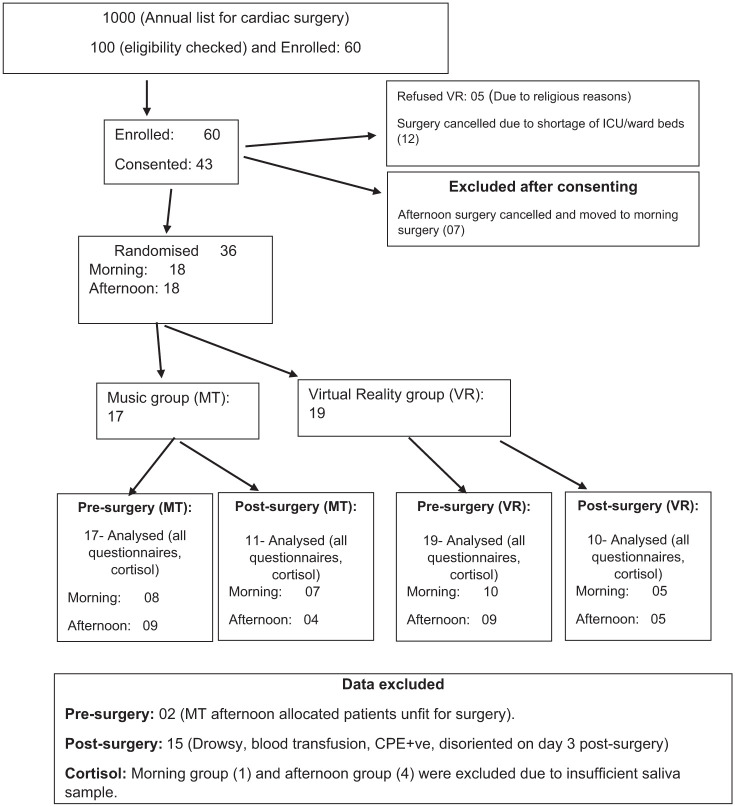
Consort diagram

A prospective, single-centre, RCT pilot with two arms, including one experimental immersive head-mounted VR group and one music group. The study was approved by the National Health Research Authority (REC reference 23/NW/0345 on 02/01/2024). Patients who provided written informed consent were randomly assigned into two groups (Figure 1). This study was undertaken at the Manchester Foundation Trust hospital and was overseen by an external steering committee, public patient involvement, and safety monitoring board.

Between March to July 2024, all elective or urgent in-patient adult cardiac surgery patients who underwent Coronary Artery Bypass Grafting (CABG) or valve or valve and CABG were included. Exclusion criteria included redo (repeat) cardiac surgery, emergency or complex cardiac surgery (aortic root surgery, aortic aneurysm, heart transplant, poor left ventricular function, triple valve surgeries, and patients with intra-aortic balloon pump in situ preoperatively), patients who did not want to listen to music, or who did not want to wear VR headsets.

Computerised randomisation was carried out by an independent statistician, and the patient allocation was revealed to the researchers (data collectors) on the morning of surgery. Data-gathering researchers, the statistician, and the cortisol analysis biochemistry team were completely blinded to the study group assignments.

## Questionnaires

The Spielberger State-Trait Anxiety Inventory (STAI) was used to score anxiety and stress level. STAI is considered as a gold standard for measuring anxiety in surgical contexts (Gürler et al 2022). It is a set of 20 statements with scores from 1 to 4 asking patients to describe how they feel ‘at this moment.’

Experience questionnaires were adapted from the Dr. VR^®^ VR Headset pilot study, conducted by the University of Bristol and Weston in July 2022, with permission. It comprised of 19 questions on different scales: four on before intervention experience, eight on after intervention experience, and seven on intervention side effects.

The Visual Analogue Scale (VAS) was used to score pain (0–5; where 0 is no pain, 5 is severe pain), relaxation (0–5; where 0 is not relaxed, 5 is fully relaxed), fatigue (0–5; where 0 is not at all, 5 is severe fatigue), and VR absorption/immersion (0–100; where 0 is not at all, 100 is fully immersed/absorbed).

## Interventions

### MT group

This group underwent the standard preoperative surgical admission process, which included hospital admission facilitated by the administrative and nursing teams, as well as consultation with the surgical team to complete preoperative assessments. Patients also received an experiential talk about the surgery pathway given by Ticker Club (heart charity) volunteers; this is an ad hoc service. Patients were provided with a perioperative booklet by preoperative outpatient nurses, which included information about the entire surgical journey from admission to discharge. The booklet covered details of the surgical procedure, anaesthesia, the intensive care unit experience, postoperative care, pain management, and the expected recovery timeline. This resource aimed to enhance patient education and reduce anxiety by improving understanding of the perioperative process. Patients were asked to listen to music of their choice for a 15 min music session with the WorWoder company wireless blue tooth headset.

**Session 1 pre-surgery:** Patients were asked to listen to the music for 15 min within 1 h of their surgery.
**Session 2 post-surgery:** Patients were asked to listen to the music post-surgery on day 3 (around 6 pm).

### VR group

**Figure 2:**
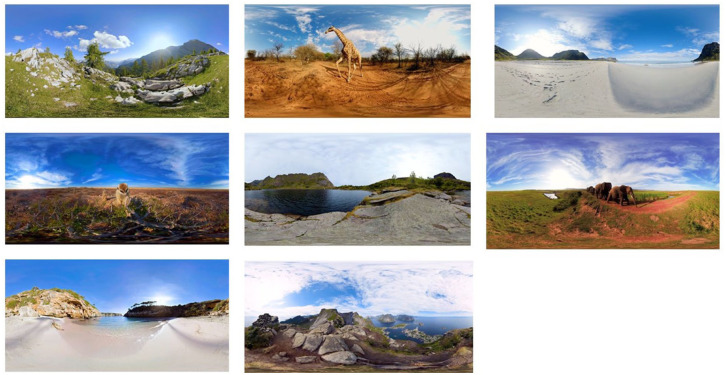
Examples of the featured virtual reality environments

This group received the same preoperative preparation as the MT group, with the addition of two VR sessions delivered via a head-mounted 3D display. This 15-min long immersive VR experience from Rescape Innovations (https://www.rescape.health/) features the visualisation of ten beautiful environments and landscapes (Figure 2).

**Session 1:** Patient received the VR session pre-surgery within 1-h time period of their surgery.
**Session 2:** They received the VR session on day 3 post-surgery (around 6 pm).

Both groups were instructed to discontinue use if they experienced any discomfort or adverse effects such as dizziness or motion sickness. These occurrences were documented as side effects and considered limitations of the respective therapies. Participants were instructed to refrain from engaging in other video game activities, watching television, or using their phones during the time of intervention to standardise the impact of their assigned intervention.

### Cortisol

Cortisol commonly known as the stress hormone is a vital hormone released by the adrenal glands in response to stress. It plays a crucial role in regulating the body’s stress response, influencing various physiological processes (Padmanabhan et al 2023). We understand that cortisol measures are complex in a range of ways, but we have assessed the feasibility and tolerability of including them in future studies to gain insight into anxiety patient-reported scores and levels produced by the patient as an objective measure for anxiety.

### Sample size and power calculation

Sample size was determined on pragmatic grounds and rule of thumb for pilot studies (Lewis et al 2021). In total, 15 patients in each group, giving a total of 30 patients, were considered practical and in line with recommendations. We aimed to enrol 10% more to compensate for dropouts or incomplete data after the surgery, making a total of 34 patients. Stratified randomisation was used to capture the equal number of patients from morning and evening to compare the stress/anxiety levels of waiting longer for surgery during the afternoon.

### Statistical analysis

Primary outcome measures, including STAI scores, and secondary outcomes such as relaxation, fatigue, and pain scores were compared before and after the interventions within each group using the paired Wilcoxon test; a non-parametric statistical method used to assess differences between two related samples when the data are not normally distributed. Changes in scores from before to after the intervention were compared between the music and VR groups using the Mann–Whitney *U*-test, a non-parametric test suitable for comparing differences between two independent groups when the data are not normally distributed. Experience and VR immersion and absorption were summarised using median and interquartile range (IQR). To assess the relationship between STAI scores and cortisol levels, the Spearman correlation coefficient was utilised.

## Results

In total, 36 participants completed pre-surgery interventions, 17 in the MT group, and 19 in the VR group. The overall median age was 66 years (IQR = 58%–73%). In total, 64% were male and 36% were female. There were 62% CABG, 28% isolated valves, 8% CABG+ valve surgery, and 2% of triple valve surgeries were performed in this cohort. All baseline preoperative characteristics, such as body mass index (BMI), smoking status, alcohol intake, diabetes, and type of surgery, were similar between the groups (Table 1). Hospital stay was similar in the two groups (MT median 17 days (IQR = 8–22) and VR median 21 days (IQR = 9–32); *p* = 0.6).

**Table 1:** Demographics of VR and music therapy

Characteristics	Overall, *N* = 36^a^	Sessions		*p* ^b^
		Music, *N* = 17^a^	VR, *N* = 19^a^	
Gender				0.14
Female	13 / 36 (36%)	4 / 17 (24%)	9 / 19 (47%)	
Male	23 / 36 (64%)	13 / 17 (76%)	10 / 19 (53%)	
Age				0.9
Median (IQR)	66 (58, 73)	65 (60, 73)	66 (58, 74)	
Range	46-79	46-79	46-79	
BMI (kg/m^2^)				0.8
Median (IQR)	27 (23, 31)	27 (26, 31)	28 (0, 32)	
Range	0-47	0-47	0-37	
Smoking				> 0.9
Never	17 / 36 (47%)	8 / 17 (47%)	9 / 19 (47%)	
Ex smoker	16 / 36 (44%)	8 / 17 (47%)	8 / 19 (42%)	
Current smoker	3 / 36 (83%)	1 / 17 (59%)	2 / 19 (11%)	
Alcohol intake				0.6
Never	8 / 35 (23%)	4 / 17 (24%)	4 / 18 (22%)	
Occasionally	14 / 35 (40%)	8 / 17 (47%)	6 / 18 (33%)	
Once a week	10 / 35 (29%)	4 / 17 (24%)	6 / 18 (33%)	
Once a day	2 / 35 (57%)	0 / 17 (0%)	2 / 18 (11%)	
Twice a day	1 / 35 (29%)	1 / 17 (59%)	0 / 18 (0%)	
Data missing	1	0	1	
Diabetes				0.049
No diabetes	22 / 36 (61%)	8 / 17 (47%)	14 / 19 (74%)	
Diet	3 / 36 (83%)	3 / 17 (18%)	0 / 19 (0%)	
Tablet	9 / 36 (25%)	6 / 17 (35%)	3 / 19 (16%)	
Insulin	2 / 36 (56%)	0 / 17 (0%)	2 / 19 (11%)	
Surgery				0.4
Elective	14 / 34 (41%)	5 / 15 (33%)	9 / 19 (47%)	
Urgent	20 / 34 (59%)	10 / 15 (67%)	10 / 19 (53%)	
Data missing	2	2	0	
Skin tissues				0.7
Clean	33 / 36 (92%)	15 / 17 (88%)	18 / 19 (95%)	
Oedematous	2 / 36 (56%)	1 / 17 (59%)	1 / 19 (53%)	
Rashes	1 / 36 (28%)	1 / 17 (59%)	0 / 19 (0%)	
Angina (CCS) scoring				0.5
None	0 / 35 (0%)	0 / 17 (0%)	0 / 18 (0%)	
Strenuous limitation	7 / 35 (20%)	2 / 17 (12%)	5 / 18 (28%)	
Slight limitation	5 / 35 (14%)	2 / 17 (12%)	3 / 18 (17%)	
Marked limitation	23 / 35 (66%)	13 / 17 (76%)	10 / 18 (56%)	
At rest or any activity	0 / 35 (0%)	0 / 17 (0%)	0 / 18 (0%)	
Data missing	1	0	1	
Stemi	7 / 32 (22%)	4 / 15 (27%)	3 / 17 (18%)	0.7
Data missing	4	2	2	
NStemi	10 / 32 (31%)	5 / 15 (33%)	5 / 17 (29%)	> 0.9
Data missing	4	2	2	
Dyspnoea (NYHA) scoring				0.4
None	9 / 36 (25%)	3 / 17 (18%)	6 / 19 (32%)	
Slight limitation	20 / 36 (56%)	9 / 17 (53%)	11 / 19 (58%)	
Marked limitation	7 / 36 (19%)	5 / 17 (29%)	2 / 19 (11%)	
At rest or any activity	0 / 36 (0%)	0 / 17 (0%)	0 / 19 (0%)	
History of headache	1 / 36 (28%)	1 / 17 (59%)	0 / 19 (0%)	0.5
History of confusion	0	0	0	—
History of skin allergies	1 / 36 (28%)	0 / 17 (0%)	1 / 19 (53%)	> 0.9
History of motion sickness	1 / 36 (28%)	0 / 17 (0%)	1 / 19 (53%)	> 0.9
History of dizziness	0	0	0	—
History of earache/discharges	0	0	0	—
Euroscore				
Median (IQR)	1.75 (1.03, 3.51)	1.61 (1.04, 3.38)	1.75 (1.01, 3.87)	0.7
Range	0.59-11.30	0.67-4.85	0.59-11.30	
Data missing	1	1	0	
Logistic Euroscore				
Median (IQR)	3.24 (2.07, 4.50)	2.71 (2.04, 4.02)	3 68 (2.31, 6.27)	0.3
Range	0.88-39.90	0.88-11.15	1.22-39.90	
Data missing	1	1	0	
Length of hospital stay				
Median (IQR)	17 (8, 30)	17 (8, 22)	21 (9, 32)	0.6
Range	5-56	6-56	5-55	
Data missing	3	3	0	

CCS: Canadian Cardiovascular Society scoring; NYHA: New York Heart Association scoring.

a Median (IQR) range or frequency (%).

b Pearson’s chi-square test; Wilcoxon rank sum test; Wilcoxon rank sum exact test; Fisher’s exact test.

## Pre-surgery

### STAI

Total STAI scores were similar between MT and VR groups before and after intervention. Both groups reported significant reduction in total individual STAI scores. MT from median 40 (IQR = 30–48) to 23 (IQR = 21–24) (*p* < 0.001) and VR from median 40 (IQR = 31–52) to 23 (IQR, 20–35) (*p* < 0.001). The change in STAI was not significantly different between MT and VR (*p* = 0.70) (Tables 2 and 3). All individual scores for each STAI category are presented in Supplementary Materials (Supplementary Table 1).

**Table 2:** STAI score change – before surgery

Characteristics	Music			VR		
	Before music *N* = 17	After music *N* = 17	*p* ^a^	Before VR *N* = 19	After VR *N* = 19	*p* ^a^
Total score			< 0.001			<0.001
Median (IQR)	40 (30, 48)	23 (21, 24)		40 (31, 52)	23 (20, 35)	
Range	21-58	20-41		20-78	20-47	
Change median (IQR)	-17.0 (-24.0, -10.0)			-15.0 (-20.0, -6.5)		*p*^b^ = 0.7

a Paired Wilcoxon signed rank test with continuity correction.

b Comparing changes between groups.

**Table 3:** STAI score – before surgery

Characteristic	Before intervention			After intervention		
	Music (*N* = 17)	VR (*N* = 19)	*p* ^a^	Music (*N* = 17)	VR (*N* = 19)	*p* ^a^
Total score			0.7			0.9
Median (IQR)	40 (30, 48)	40 (31, 52)		23 (21, 24)	23 (20, 35)	
Range	21-58	20-78		20-41	20-47	

a Wilcoxon rank sum test.

### User experience

Patients were asked to complete an experience questionnaire before and after intervention. The two groups were similar in different experiences before their interventions. Overall median (IQR) scores for their feeling (score 0–3) were 2.00 (2.00–3.00) (*p* > 0.90), stress level (score 0–3) 1.50 (1.00–2.00) (*p* = 0.80) and calm (score 0–3) 1.00 (1.00–2.00) (*p* = 0.80). Both groups were also similar in experiences after intervention in relation to felt relaxed (score 0–4), the overall median (IQR) scores were 4.00 (3.00–4.00) (*p* = 0.2), felt less stressed (score 0–4) 3.00 (3.00–4.00) (*p* = 0.3), and calm (score 0–4) 3.00 (3.00–4.00) (*p* = 0.5). The side effects of using the headsets for these interventions, such as felt uncomfortable, nausea and vomiting, dizziness, claustrophobia, skin irritation, and headache, were not significantly different between the two groups (Supplementary Tables 2 and 2a).

### Absorption and immersion

Eighteen patients completed the absorption and immersion ratings for VR session, with participants reporting a median absorption score of 90 (IQR = 80–94) and a median immersion score of 90 (IQR = 80–90).

### Physiological parameters

*Before intervention:* Physiological parameters before intervention were similar in the two groups (Supplementary Table 3). The overall median for heart rate was 65 (IQR, 61–71) versus 74 (IQR, 68–80) for MT versus VR, respectively; *p* = 0.077; systolic blood pressure (BP) was 128 (IQR, 109–142) versus 126 (IQR, 109–138) for MT versus VR, respectively; *p* = 0.6; diastolic BP was 76 (IQR, 66–84) versus 79 (IQR, 68–85) for MT versus VR, respectively; *p* = 0.7; respiratory rate was 17 (IQR = 16–17) versus 17 (IQR, 16–17) for MT versus VR, respectively; *p* = 0.6; and SpO_2_ was 97 (IQR, 96–97) versus 97 (IQR, 96–97) for MT versus VR, respectively; *p* = 0.7.

*After intervention:* The overall median for heart rate was 70 (IQR, 62–76) versus 79 (IQR, 70–87) for MT versus VR, respectively; *p* = 0.087; systolic BP was 125 (IQR, 115–135) versus 129 (IQR, 110–137) for MT versus VR, respectively; *p* > 0.9; diastolic BP was 75 (IQR, 66–83) versus 76 (IQR, 67–81) for MT versus VR, respectively; *p* = 0.9; respiratory rate was 17 (IQR, 16–17) versus 17 (IQR, 16–18) for MT versus VR, respectively; *p* > 0.9; and SpO_2_ was 97 (IQR, 96–98) versus 97 (IQR, 96–98) for MT versus VR, respectively; *p* = 0.8 (Supplementary Tables 3 and 3a).

*Relaxation scores:* Overall median scores for relaxation before intervention were 3.00 (IQR, 0.00–3.25) versus 1.00 (IQR, 1.00–2.50) for MT versus VR, respectively; *p* = 0.4. After intervention, overall median score was 3.00 (IQR, 0.00–3.25) versus 3.50 (IQR, 1.50–4.75) for MT versus VR, respectively; *p* = 0.4.

*Pain and fatigue score:* No difference was noted on either pain or fatigue scores between the two groups. Before intervention, median pain score was 0.00 (IQR, 0.00–0.00) versus 0.00 (IQR, 0.00–0.00) for MT versus VR, respectively; *p* = 0.9. After intervention, overall median score was 0.00 (IQR, 0.00–0.00) versus 0.50 (IQR, 0.00–1.50) for MT versus VR, respectively; *p* = 0.2. Similarly, overall median fatigue score before intervention was 1.00 (IQR, 0.00–1.25) versus 1.00 (IQR, 0.25–2.00) for MT versus VR respectively; *p* = 0.7. After intervention, median fatigue score was 1.00 (0.00–1.00) versus 1.00 (IQR, 0.00–1.00) for MT versus VR, respectively; *p* > 0.9.

*Cortisol analysis:* There was no change noted on cortisol levels in either group. The median cortisol level for MT was 6.0 (IQR, 4.8–7.6) to 5.6 (IQR, 4.1–7.7) (*p* = 0.2) and for VR was 4.45 (IQR, 3.05–8.53) to 4.45 (IQR, 2.73–6.83) (*p* = 0.11). The change in cortisol level was not significantly different between the two groups (Supplementary Table 4).

## Post-surgery

A total of 15 patients (MT-06 and VR-09) were unable to complete the interventions post-surgery. The main reasons that patients were unable to complete the interventions on day 3 were due to: drowsiness, confusion, disorientation, on blood transfusion, and requirement of oxygen mask. One patient contracted Carbapenemase-Producing Enterobacteriaceae (CPE) post-surgery. In the VR group, only eight patients had STAI-completed scores for both before and after intervention.

### STAI

Both music and VR group patients reported similar reduction in total STAI scores before and after intervention post-surgery. The median score for MT was reduced from before intervention 31 (IQR, 26–32) to after intervention 23 (IQR, 20–27) (*p* < 0.005). Likewise, the median scores for the VR group before intervention were 33 (IQR, 23–41) to after intervention 24 (IQR, 22–30) (*p* = 0.059) (Tables 4 and 5). There was no difference between the two groups.

**Table 4:** STAI score change – after surgery

Characteristic	Music			VR		
	Before music *N* = 11	After music *N* = 11	*p* ^a^	Before VR *N* = 10	After VR *N* = 8	*p* ^a^
Total score			<0.005			0.059
Median (IQR)	31 (26, 32)	23 (20, 27)		33 (23, 41)	24 (22, 30)	
Range	22-47	20-32		20-44	20-40	
Change median (IQR)	-8.0 (-13.5, -2.0)			-5.0 (-8.3, 0.0)		*p*^b^ = 0.3

a Paired Wilcoxon signed rank test with continuity correction.

b Comparing changes between groups.

**Table 5:** STAI score – after surgery

	Before intervention			After intervention		
Characteristic	Music *N* = 11	VR *N* = 10	*p* ^a^	Music *N* = 11	VR *N* = 8	*p* ^a^
Total score			0.8			0.9
Median (IQR)	31 (26, 32)	33 (23, 41)		23 (20, 27)	24 (22, 30)	

a Wilcoxon rank sum test.

### User experience

Patients were asked to rate their experience before and after intervention after surgery. The experience was similar between the two groups. Prior to starting the session, overall median scores for their feeling were 3.00 (IQR, 2.25–3.00), stress level was 1.00 (IQR, 0.00–2.00), and calmness was 1.00 (IQR, 1.00–2.00). Similar patterns were observed after intervention in relation to feeling relaxed overall median was 4.00 (IQR, 3.00–4.00), felt less stressed was 4.00 (IQR, 3.00–4.00), and felt calmer was 3.00 (IQR, 3.00–4.00) (Supplementary Table 5).

### Cortisol analysis

The salivary cortisol levels are expressed in nmol/L. Levels were very similar before and after intervention in both groups. The median level for MT was 5.0 (IQR, 2–7) to 5.0 (IQR, 3–5). Similarly, VR group level was 4.1 (2.8–8.3) to 4.4 (4.0–7.5) (*p* = 0.3) (Supplementary Table 4).

## Discussion

Our study results show that before surgery, there is no significant difference between the MT group and VR group before (*p* = 0.7) and after intervention (*p* = 0.9). In both the MT and VR groups, patients reported a significant reduction of total STAI scores after intervention (*p* < 0.001). For cardiac surgery patients, both interventions have shown significant promise in this study. However, we found that not all patients are suitable for VR interventions. We had five patients’ refusal to the VR device due to preferred religious contexts that were not part of the current software programme.

An immersive VR experience may help patients visualise and interact with a calming, controlled environment, which reduces anxiety levels. Hendricks et al (2020) found that cardiac surgery patients who used VR reported significant reduction in Spielberger State-Trait anxiety scores compared to those who received standard care. These findings suggest that VR can be an effective non-pharmacological tool for managing preoperative anxiety in high-stress surgical procedures. Similar results were seen in our study; most patients felt calmer (score > 2).

Our study assessed the feasibility of recruiting more diverse populations to see the acceptance rate, and there were 40% British Asian (Indian, Pakistani, Chinese), 40% white Caucasian, and 20% African descents. The 5% of patients who refused to enrol were British Pakistani and Filipino and they would have preferred to hear spiritual songs or be immersed in spiritual settings. This study explored the use of VR technology to reduce the anxiety and stress prior to cardiac surgery. More diverse enrolment of non-representative populations and addressing their needs will reduce health disparities (Bradt et al 2013) and provide equal access to technology which targets a reduction in anxiety and stress.

## Limitations

A primary limitation was the absence of a control standard practice group for comparison with the VR and MT groups in both the assessment of STAI scores and cortisol levels. To mitigate this, we employed before and after intervention comparisons within each group, used validated outcome measures, and applied consistent intervention protocols and strict participant selection criteria. In addition, we collected feasibility and acceptability data to support the interpretation of findings. While the lack of a control group limits causal inference, these strategies helped strengthen the internal validity of this pilot study and will inform the design of future controlled trials. Following the conduct of the study, we have concluded that selecting postoperative day 3 for the VR or MT was not ideal from the perspective of completing the assessments that were required. We found that a significant proportion of the participants were affected by strong analgesia or postoperative fatigue on the third post-surgery day, and we would suggest day 5 for assessment for future studies. In addition, the study design would have benefitted from qualitative interviews with each participant to gain insights into their perspectives on the intervention.

## Conclusion

VR immersive-guided mindfulness and MT can be effective techniques to reduce preoperative anxiety in patients undergoing cardiac surgery. Our findings suggest that both techniques can be utilised in the clinical settings prior to surgery. We strongly recommend that future studies conduct their interventions and assessments on day 5, rather than on day 3, to obtain more consistent assessment. On day 3, patients are often still in the process of weaning off stronger analgesia and may be fatigued, which can affect assessment. We would recommend further studies with larger sample sizes to validate these findings.

## Supplemental Material

sj-docx-1-ppj-10.1177_17504589251370291 – Supplemental material for A randomised pilot study evaluating music therapy and virtual reality mindfulness sessions for reducing anxiety and stress in patients undergoing first-time elective cardiac surgery Supplemental material, sj-docx-1-ppj-10.1177_17504589251370291 for A randomised pilot study evaluating music therapy and virtual reality mindfulness sessions for reducing anxiety and stress in patients undergoing first-time elective cardiac surgery by Bhuvaneswari Krishnamoorthy, Moslem Abdelghafar, Rick Air, Joshua Halyckyj-Smith, Angelo Citarella, Edward McLaughlin, Heather Iles-Smith, Rebecca Elliott, James Barnard, Azita Rajai and Rajamiyer Venkateswaran in Journal of Perioperative Practice

sj-docx-2-ppj-10.1177_17504589251370291 – Supplemental material for A randomised pilot study evaluating music therapy and virtual reality mindfulness sessions for reducing anxiety and stress in patients undergoing first-time elective cardiac surgery Supplemental material, sj-docx-2-ppj-10.1177_17504589251370291 for A randomised pilot study evaluating music therapy and virtual reality mindfulness sessions for reducing anxiety and stress in patients undergoing first-time elective cardiac surgery by Bhuvaneswari Krishnamoorthy, Moslem Abdelghafar, Rick Air, Joshua Halyckyj-Smith, Angelo Citarella, Edward McLaughlin, Heather Iles-Smith, Rebecca Elliott, James Barnard, Azita Rajai and Rajamiyer Venkateswaran in Journal of Perioperative Practice

sj-docx-3-ppj-10.1177_17504589251370291 – Supplemental material for A randomised pilot study evaluating music therapy and virtual reality mindfulness sessions for reducing anxiety and stress in patients undergoing first-time elective cardiac surgery Supplemental material, sj-docx-3-ppj-10.1177_17504589251370291 for A randomised pilot study evaluating music therapy and virtual reality mindfulness sessions for reducing anxiety and stress in patients undergoing first-time elective cardiac surgery by Bhuvaneswari Krishnamoorthy, Moslem Abdelghafar, Rick Air, Joshua Halyckyj-Smith, Angelo Citarella, Edward McLaughlin, Heather Iles-Smith, Rebecca Elliott, James Barnard, Azita Rajai and Rajamiyer Venkateswaran in Journal of Perioperative Practice

sj-docx-4-ppj-10.1177_17504589251370291 – Supplemental material for A randomised pilot study evaluating music therapy and virtual reality mindfulness sessions for reducing anxiety and stress in patients undergoing first-time elective cardiac surgery Supplemental material, sj-docx-4-ppj-10.1177_17504589251370291 for A randomised pilot study evaluating music therapy and virtual reality mindfulness sessions for reducing anxiety and stress in patients undergoing first-time elective cardiac surgery by Bhuvaneswari Krishnamoorthy, Moslem Abdelghafar, Rick Air, Joshua Halyckyj-Smith, Angelo Citarella, Edward McLaughlin, Heather Iles-Smith, Rebecca Elliott, James Barnard, Azita Rajai and Rajamiyer Venkateswaran in Journal of Perioperative Practice

sj-docx-5-ppj-10.1177_17504589251370291 – Supplemental material for A randomised pilot study evaluating music therapy and virtual reality mindfulness sessions for reducing anxiety and stress in patients undergoing first-time elective cardiac surgery Supplemental material, sj-docx-5-ppj-10.1177_17504589251370291 for A randomised pilot study evaluating music therapy and virtual reality mindfulness sessions for reducing anxiety and stress in patients undergoing first-time elective cardiac surgery by Bhuvaneswari Krishnamoorthy, Moslem Abdelghafar, Rick Air, Joshua Halyckyj-Smith, Angelo Citarella, Edward McLaughlin, Heather Iles-Smith, Rebecca Elliott, James Barnard, Azita Rajai and Rajamiyer Venkateswaran in Journal of Perioperative Practice

sj-docx-6-ppj-10.1177_17504589251370291 – Supplemental material for A randomised pilot study evaluating music therapy and virtual reality mindfulness sessions for reducing anxiety and stress in patients undergoing first-time elective cardiac surgery Supplemental material, sj-docx-6-ppj-10.1177_17504589251370291 for A randomised pilot study evaluating music therapy and virtual reality mindfulness sessions for reducing anxiety and stress in patients undergoing first-time elective cardiac surgery by Bhuvaneswari Krishnamoorthy, Moslem Abdelghafar, Rick Air, Joshua Halyckyj-Smith, Angelo Citarella, Edward McLaughlin, Heather Iles-Smith, Rebecca Elliott, James Barnard, Azita Rajai and Rajamiyer Venkateswaran in Journal of Perioperative Practice

sj-docx-7-ppj-10.1177_17504589251370291 – Supplemental material for A randomised pilot study evaluating music therapy and virtual reality mindfulness sessions for reducing anxiety and stress in patients undergoing first-time elective cardiac surgery Supplemental material, sj-docx-7-ppj-10.1177_17504589251370291 for A randomised pilot study evaluating music therapy and virtual reality mindfulness sessions for reducing anxiety and stress in patients undergoing first-time elective cardiac surgery by Bhuvaneswari Krishnamoorthy, Moslem Abdelghafar, Rick Air, Joshua Halyckyj-Smith, Angelo Citarella, Edward McLaughlin, Heather Iles-Smith, Rebecca Elliott, James Barnard, Azita Rajai and Rajamiyer Venkateswaran in Journal of Perioperative Practice
