# Supplementary material for: A randomised pilot study evaluating music therapy and virtual reality mindfulness sessions for reducing anxiety and stress in patients undergoing first-time elective cardiac surgery
Source: J Perioper Pract. 2025 Oct 4;36(1-2):59–67. doi: 10.1177/17504589251370291 (PMC12712224; doi:10.1177/17504589251370291)
Supplement: sj-docx-1-ppj-10.1177_17504589251370291 – Supplemental material for A randomised pilot study evaluating music therapy and virtual reality mindfulness sessions for reducing anxiety and stress in patients undergoing first-time elective cardiac surgery [file sj-docx-1-ppj-10.1177_17504589251370291.docx]

**Supplementary table 1-Pre intervention and Post intervention scores for individual questions (anxiety absent scores were reversed back)**

|  | **Music** | | | **VR** | | |
| --- | --- | --- | --- | --- | --- | --- |
| **Characteristic** | **Pre-Music, N = 17^1^** | **Post-Music, N = 17^1^** | **p-value^2^** | **Pre-VR, N = 19^1^** | **Post-VR, N = 19^1^** | **p-value^2^** |
| Total score | 40(30,48) | 23(21,24) | <0.001 | 40(31,52) | 23(20,35) | <0.001 |
| tense | 3.0(2.0,4.0) | 1.0(1.0,2.0) | 0.002 | 2.0(1.0,3.0) | 1.0(1.0,1.50) | 0.005 |
| strained | 2.0(1.0,2.0) | 1.0(1.0,1.0) | 0.012 | 2.0(1.0,3.0) | 1.0(1.0,1.0) | 0.003 |
| upset | 1.0(1.0,2.0) | 1.0(1.0,1.0) | 0.010 | 1.0(1.0,2.0) | 1.0(1.0,1.0) | 0.036 |
| worrying over possible misfortunes | 1.0(1.0,1.0) | 1.0(1.0,1.0) | 0.089 | 2.0(1.50,4.0) | 1.0(1.0,2.0) | 0.003 |
| frightened | 2.0(1.0,3.0) | 1.0(1.0,1.0) | 0.005 | 2.0(1.50,3.0) | 1.0(1.0,2.0) | 0.004 |
| uncomfortable | 1.0(1.0,2.0) | 1.0(1.0,1.0) | 0.7 | 1.0(1.0,2.0) | 1.0(1.0,1.0) | 0.013 |
| nervous | 3.0(2.0,4.0) | 1.0(1.0,2.0) | 0.002 | 3.0(2.0,3.0) | 1.0(1.0,2.0) | <0.001 |
| jittery | 2.0(1.0,2.0) | 1.0(1.0,1.0) | 0.021 | 2.0(1.0,3.0) | 1.0(1.0,1.0) | 0.005 |
| indecisive | 1.0(1.0,1.0) | 1.0(1.0,1.0) | >0.9 | 1.0(1.0,1.50) | 1.0(1.0,1.0) | 0.054 |
| worried | 2.0(1.0,4.0) | 1.0(1.0,1.0) | 0.005 | 3.0(2.0,3.0) | 1.0(1.0,2.0) | 0.003 |
| confused | 1.0(1.0,1.0) | 1.0(1.0,1.0) | >0.9 | 1.0(1.0,1.0) | 1.0(1.0,1.0) | 0.3 |
| calm | 3.0(2.0,4.0) | 4.0(4.0,4.0) | 0.005 | 3.0(2.0,3.50) | 4.0(3.0,4.0) | 0.002 |
| secure | 3.0(3.0,4.0) | 4.0(4.0,4.0) | 0.004 | 3.0(3.0,4.0) | 4.0(3.0,4.0) | 0.025 |
| at ease | 3.0(2.0,3.0) | 4.0(4.0,4.0) | 0.005 | 3.0(2.50,3.50) | 4.0(3.0,4.0) | 0.003 |
| satisfied | 3.0(2.0,3.0) | 4.0(4.0,4.0) | 0.012 | 3.0(2.0,4.0) | 4.0(3.0,4.0) | 0.003 |
| self-confident | 3.0(2.0,4.0) | 4.0(4.0,4.0) | 0.007 | 3.0(2.0,4.0) | 4.0(3.0,4.0) | 0.040 |
| relaxed | 3.0(3.0,4.0) | 4.0(4.0,4.0) | 0.004 | 2.0(2.0,3.0) | 4.0(3.0,4.0) | 0.002 |
| content | 3.0(2.0,4.0) | 4.0(4.0,4.0) | 0.003 | 2.0(1.50,4.0) | 4.0(3.0,4.0) | 0.002 |
| steady | 4.0(3.0,4.0) | 4.0(4.0,4.0) | 0.026 | 3.0(2.50,4.0) | 4.0(3.0,4.0) | 0.031 |
| pleasant | 4.0(4.0,4.0) | 4.0(4.0,4.0) | 0.095 | 3.0(2.0,4.0) | 4.0(3.0,4.0) | 0.019 |
| ^1^Median (IQR) or Frequency (%)  ^2^Paired Wilcox test | | | | | | |
|  | | | | | | |
