# Supplementary material for: A randomised pilot study evaluating music therapy and virtual reality mindfulness sessions for reducing anxiety and stress in patients undergoing first-time elective cardiac surgery
Source: J Perioper Pract. 2025 Oct 4;36(1-2):59–67. doi: 10.1177/17504589251370291 (PMC12712224; doi:10.1177/17504589251370291)
Supplement: sj-docx-2-ppj-10.1177_17504589251370291 – Supplemental material for A randomised pilot study evaluating music therapy and virtual reality mindfulness sessions for reducing anxiety and stress in patients undergoing first-time elective cardiac surgery [file sj-docx-2-ppj-10.1177_17504589251370291.docx]

**Supplementary table 2- VR/Music Experience Before Surgery**

| **Characteristic** | **Music**  N = 17^1^ | **VR**,  N = 19^1^ |
| --- | --- | --- |
| How did you felt before starting the headset session |  |  |
| Median (IQR) | 2.00 (2.00, 3.00) | 2.00 (2.00, 3.00) |
| Minimum-Maximum | 2.00-5.00 | 2.00-5.00 |
| How stressed are you before starting the headset session |  |  |
| Median (IQR) | 2.00 (1.00, 2.00) | 1.00 (1.00, 2.00) |
| Minimum-Maximum | 0.00-2.00 | 0.00-3.00 |
| Before using the headset session, how much time do you allocate to priorities self-care/looking after yourself? |  |  |
| Never | 1 (5.9%) | 2 (11%) |
| Once a month | 2 (12%) | 2 (11%) |
| Once a week | 3 (18%) | 4 (21%) |
| Twice a week | 2 (12%) | 2 (11%) |
| Once a day | 2 (12%) | 0 (0%) |
| 1 hour a day | 2 (12%) | 7 (37%) |
| Half a day | 5 (29%) | 2 (11%) |
| How calm you are before using the headset session |  |  |
| Median (IQR) | 1.00 (1.00, 2.00) | 1.00 (1.00, 2.00) |
| Minimum-Maximum | 0.00-3.00 | 0.00-3.00 |
| Using the headset session was a pleasurable experience |  |  |
| Median (IQR) | 4.00 (3.00, 4.00) | 3.00 (3.00, 4.00) |
| Range | 2.00-4.00 | 3.00-4.00 |
| After using the headset session, I felt relaxed |  |  |
| Median (IQR) | 4.00 (3.00, 4.00) | 3.00 (3.00, 4.00) |
| Range | 2.00-4.00 | 2.00-4.00 |
| After using the headset session, I felt less stressed compared to before attending the session |  |  |
| Median (IQR) | 4.00 (3.00, 4.00) | 3.00 (3.00, 4.00) |
| Range | 1.00-4.00 | 1.00-4.00 |
| After using the headset session, I felt calmer |  |  |
| Median (IQR) | 3.00 (3.00, 4.00) | 3.00 (3.00, 4.00) |
| Range | 2.00-4.00 | 1.00-4.00 |
| Using the headset enhanced my mood |  |  |
| Median (IQR) | 3.00 (3.00, 4.00) | 3.00 (3.00, 4.00) |
| Range | 2.00-4.00 | 1.00-4.00 |
| Using the headset session made me to think about doing more to priorities self-care |  |  |
| Median (IQR) | 3.00 (3.00, 4.00) | 3.00 (2.00, 3.50) |
| Range | 1.00-4.00 | 1.00-4.00 |
| Using the headset made me to feel uncomfortable/ uneasy |  |  |
| Median (IQR) | 0.00 (0.00, 0.00) | 0.00 (0.00, 1.00) |
| Range | 0.00-3.00 | 0.00-4.00 |
| Using the headset made me to feel vomiting |  |  |
| Median (IQR) | 0.00 (0.00, 0.00) | 0.00 (0.00, 0.50) |
| Range | 0.00-1.00 | 0.00-1.00 |
| Using the headset made me to feel dizzy (lightheaded) |  |  |
| Median (IQR) | 0.00 (0.00, 0.00) | 0.00 (0.00, 0.50) |
| Range | 0.00-1.00 | 0.00-1.00 |
| Using the headset made me to feel nausea |  |  |
| Median (IQR) | 0.00 (0.00, 0.00) | 0.00 (0.00, 0.50) |
| Range | 0.00-1.00 | 0.00-1.00 |
| Using the headset made me to feel claustrophobia |  |  |
| Median (IQR) | 0.00 (0.00, 0.00) | 0.00 (0.00, 1.00) |
| Range | 0.00-3.00 | 0.00-1.00 |
| Using the headset made me to itch my facial skin |  |  |
| Median (IQR) | 0.00 (0.00, 0.00) | 0.00 (0.00, 1.00) |
| Range | 0.00-1.00 | 0.00-1.00 |
| Using the headset made me to feel headache |  |  |
| Median (IQR) | 0.00 (0.00, 0.00) | 0.00 (0.00, 0.50) |
| Range | 0.00-3.00 | 0.00-1.00 |
| Using the headset was fun and cool |  |  |
| Median (IQR) | 4.00 (3.00, 4.00) | 4.00 (3.00, 4.00) |
| Range | 2.00-4.00 | 2.00-4.00 |
| I would strongly recommend future patients to use headset before going to surgery |  |  |
| Median (IQR) | 4.00 (4.00, 4.00) | 4.00 (3.00, 4.00) |
| Range | 3.00-4.00 | 2.00-4.00 |
| ^1^Median (IQR) Range or Frequency (%) | | |
