# Supplementary material for: A randomised pilot study evaluating music therapy and virtual reality mindfulness sessions for reducing anxiety and stress in patients undergoing first-time elective cardiac surgery
Source: J Perioper Pract. 2025 Oct 4;36(1-2):59–67. doi: 10.1177/17504589251370291 (PMC12712224; doi:10.1177/17504589251370291)
Supplement: sj-docx-3-ppj-10.1177_17504589251370291 – Supplemental material for A randomised pilot study evaluating music therapy and virtual reality mindfulness sessions for reducing anxiety and stress in patients undergoing first-time elective cardiac surgery [file sj-docx-3-ppj-10.1177_17504589251370291.docx]

**Supplementary table 2a- VR and MT Experience scores before Surgery**

| **Characteristic** | | **Overall** | **Music**  N = 17^1^ | **VR**,  N = 19^1^ | **p-value^2^** |
| --- | --- | --- | --- | --- | --- |
| How did you felt before starting the headset session | |  |  |  | >0.9 |
| Median (IQR) | | 2.00 (2.00, 3.00) | 2.00 (2.00, 3.00) | 2.00 (2.00, 3.00) |  |
| Minimum-Maximum | | 2.00-5.00 | 2.00-5.00 | 2.00-5.00 |  |
| How stressed are you before starting the headset session | |  |  |  |  |
| Median (IQR) | | 1.50 (1.00, 2.00) | 2.00 (1.00, 2.00) | 1.00 (1.00, 2.00) | 0.8 |
| Minimum-Maximum | | 0.00-3.00 | 0.00-2.00 | 0.00-3.00 |  |
| Before using the headset session, how much time do you allocate to priorities self-care/looking after yourself? | |  |  |  | 0.4 |
| Never | | 3(8.3%) | 1 (5.9%) | 2 (11%) |  |
| Once a month | | 4(11%) | 2 (12%) | 2 (11%) |  |
| Once a week | | 7(19%) | 3 (18%) | 4 (21%) |  |
| Twice a week | | 4(11%) | 2 (12%) | 2 (11%) |  |
| Once a day | | 2(5.6%) | 2 (12%) | 0 (0%) |  |
| 1 hour a day | | 9(25%) | 2 (12%) | 7 (37%) |  |
| Half a day | | 7(19%) | 5 (29%) | 2 (11%) |  |
| How calm you are before using the headset session | |  |  |  |  |
| Median (IQR) | | 1.00 (1.00, 2.00) | 1.00 (1.00, 2.00) | 1.00 (1.00, 2.00) | 0.8 |
| Minimum-Maximum | | 0.00-3.00 | 0.00-3.00 | 0.00-3.00 |  |
| Using the headset session was a pleasurable experience | |  |  |  |  |
| Median (IQR) | | 4.00 (3.00, 4.00) | 4.00 (3.00, 4.00) | 3.00 (3.00, 4.00) | 0.2 |
| Range | | 2.00-4.00 | 2.00-4.00 | 3.00-4.00 |  |
| After using the headset session, I felt relaxed | |  |  |  | 0.2 |
| Median (IQR) | | 4.00 (3.00, 4.00) | 4.00 (3.00, 4.00) | 3.00 (3.00, 4.00) |  |
| Range | | 2.00-4.00 | 2.00-4.00 | 2.00-4.00 |  |
| After using the headset session, I felt less stressed compared to before attending the session | |  |  |  | 0.3 |
| Median (IQR) | | 3.00 (3.00, 4.00) | 4.00 (3.00, 4.00) | 3.00 (3.00, 4.00) |  |
| Range | | 1.00-4.00 | 1.00-4.00 | 1.00-4.00 |  |
| After using the headset session, I felt calmer | |  |  |  | 0.5 |
| Median (IQR) | | 3.00 (3.00, 4.00) | 3.00 (3.00, 4.00) | 3.00 (3.00, 4.00) |  |
| Range | | 1.00-4.00 | 2.00-4.00 | 1.00-4.00 |  |
| Using the headset enhanced my mood | |  |  |  | 0.9 |
| Median (IQR) | | 3.00 (3.00, 4.00) | 3.00 (3.00, 4.00) | 3.00 (3.00, 4.00) |  |
| Range | | 1.00-4.00 | 2.00-4.00 | 1.00-4.00 |  |
| Using the headset session made me to think about doing more to priorities self-care | |  |  |  | 0.2 |
| Median (IQR) | | 3.00 (2.00, 4.00) | 3.00 (3.00, 4.00) | 3.00 (2.00, 3.50) |  |
| Range | | 1.00-4.00 | 1.00-4.00 | 1.00-4.00 |  |
| Using the headset made me to feel uncomfortable/ uneasy | |  |  |  | 0.4 |
| Median (IQR) | | 0.00 (0.00, 1.00) | 0.00 (0.00, 0.00) | 0.00 (0.00, 1.00) |  |
| Range | | 0.00-4.00 | 0.00-3.00 | 0.00-4.00 |  |
| Using the headset made me to feel vomiting | |  |  |  | 0.3 |
| Median (IQR) | | 0.00 (0.00, 0.00) | 0.00 (0.00, 0.00) | 0.00 (0.00, 0.50) |  |
| Range | | 0.00-1.00 | 0.00-1.00 | 0.00-1.00 |  |
| Using the headset made me to feel dizzy (lightheaded) | |  |  |  | 0.3 |
| Median (IQR) | | 0.00 (0.00, 0.00) | 0.00 (0.00, 0.00) | 0.00 (0.00, 0.50) |  |
| Range | | 0.00-1.00 | 0.00-1.00 | 0.00-1.00 |  |
| Using the headset made me to feel nausea | |  |  |  | 0.3 |
| Median (IQR) | | 0.00 (0.00, 0.00) | 0.00 (0.00, 0.00) | 0.3 |  |
| Range | | 0.00-1.00 | 0.00-1.00 |  |  |
| Using the headset made me to feel claustrophia | |  |  |  | 0.4 |
| Median (IQR) | | 0.00 (0.00, 0.25) | 0.00 (0.00, 0.00) | 0.4 |  |
| Range | | 0.00-3.00 | 0.00-3.00 |  |  |
| Using the headset made me to itch my facial skin | |  |  |  | 0.4 |
| Median (IQR) | | 0.00 (0.00, 0.25) | 0.00 (0.00, 0.00) | 0.4 |  |
| Range | | 0.00-1.00 | 0.00-1.00 |  |  |
| Using the headset made me to feel headache | |  |  |  | 0.14 |
| Median (IQR) | | 0.00 (0.00, 0.00) | 0.00 (0.00, 0.00) | 0.14 |  |
| Range | | 0.00-3.00 | 0.00-3.00 |  |  |
| Using the headset was fun and cool | |  |  |  | 0.6 |
| Median (IQR) | | 4.00 (3.00, 4.00) | 4.00 (3.00, 4.00) | 0.6 |  |
| Range | | 2.00-4.00 | 2.00-4.00 |  |  |
| I would strongly recommend future patients to use headset before going to surgery | |  |  |  | 0.4 |
| Median (IQR) | | 4.00 (3.00, 4.00) | 4.00 (4.00, 4.00) | 0.4 |  |
| Range | | 2.00-4.00 | 3.00-4.00 | 2.00-4.00 |  |
|  | ^1^Median (IQR) Range or Frequency (%)  ^2^Wilcoxon rank sum test; Fisher's exact test | | | |  |

**VR Immersion and Absorption Before Surgery^1^**

| **Characteristic** | **N = 19** |
| --- | --- |
| Absorption |  |
| Median (IQR) | 90 (80, 94) |
| Minimum-Maximum | 10-100 |
| Unknown | 1 |
| Immersion |  |
| Median (IQR) | 90 (80, 90) |
| Minimum-Maximum | 10-100 |
| Unknown | 1 |
|  |  |

^1^ Two from Music group were

excluded from immersion and

absorption data
