# Supplementary material for: A randomised pilot study evaluating music therapy and virtual reality mindfulness sessions for reducing anxiety and stress in patients undergoing first-time elective cardiac surgery
Source: J Perioper Pract. 2025 Oct 4;36(1-2):59–67. doi: 10.1177/17504589251370291 (PMC12712224; doi:10.1177/17504589251370291)
Supplement: sj-docx-4-ppj-10.1177_17504589251370291 – Supplemental material for A randomised pilot study evaluating music therapy and virtual reality mindfulness sessions for reducing anxiety and stress in patients undergoing first-time elective cardiac surgery [file sj-docx-4-ppj-10.1177_17504589251370291.docx]

**Supplementary table 3- VR and MT Physiology- Before Surgery**

|  | **Music** | | | **VR** | | |
| --- | --- | --- | --- | --- | --- | --- |
| **Characteristic** | **Pre-Music**, N = 17^1^ | **Post-Music**, N = 17^1^ | **p-value**^2^ | **Pre-VR**, N = 19^1^ | **Post-VR**, N = 19^1^ | **p-value**^2^ |
| Heartrate |  |  | 0.6 |  |  | 0.3 |
| Median (IQR) | 65 (61, 71) | 70 (62, 76) |  | 74 (68, 80) | 79 (70, 87) |  |
| Minimum-Maximum | 54-88 | 52-92 |  | 58-90 | 59-99 |  |
| Unknown |  |  |  | 1 | 0 |  |
| Systolic Bp |  |  | 0.14 |  |  | 0.8 |
| Median (IQR) | 128 (109, 142) | 125 (115, 135) |  | 126 (109, 138) | 129 (110, 137) |  |
| Minimum-Maximum | 91-168 | 100-152 |  | 88-173 | 94-146 |  |
| Unknown |  |  |  | 1 | 0 |  |
| Diastolic Bp |  |  | >0.9 |  |  | 0.6 |
| Median (IQR) | 76 (66, 84) | 75 (66, 83) |  | 79 (68, 85) | 76 (67, 81) |  |
| Minimum-Maximum | 55-102 | 62-98 |  | 55-91 | 59-92 |  |
| Unknown |  |  |  | 1 | 0 |  |
| Respiratory rate |  |  | 0.3 |  |  | >0.9 |
| Median (IQR) | 17 .0 (16 .0, 17 .0) | 17 .0 (16 .0, 17 .0) |  | 17 .0 (16 .0, 17 .0) | 17 .0 (16 .0, 18 .0) |  |
| Minimum-Maximum | 15 .0-18 .0 | 16 .0-18 .0 |  | 15 .0-19 .0 | 15 .0-18 .0 |  |
| Unknown |  |  |  | 1 | 0 |  |
| SPO2 |  |  | 0.3 |  |  | 0.4 |
| Median (IQR) | 97 .0 (96 .0, 97 .0) | 97 .0 (96 .0, 98 .0) |  | 97 .0 (96 .0, 97 .0) | 97 .0 (96 .0, 98 .0) |  |
| Minimum-Maximum | 95 .0-99 .0 | 95 .0-100 .0 |  | 94 .0-98 .0 | 93 .0-99 .0 |  |
| Unknown |  |  |  | 1 | 0 |  |
| Pain score |  |  | >0.9 |  |  |  |
| Median (IQR) | 0 .0 (0 .0, 0 .0) | 0 .0 (0 .0, 0 .0) |  | 0 .0 (0 .0, 0 .0) | 0.50 (0 .0, 1.50) |  |
| Minimum-Maximum | 0 .0-1 .0 | 0 .0-1 .0 |  | 0 .0-3 .0 | 0 .0-3 .0 |  |
| Unknown | 6 | 9 |  | 9 | 15 |  |
| Fatigue scale |  |  | >0.9 |  |  |  |
| Median (IQR) | 1 .0 (0 .0, 1.25) | 1 .0 (0 .0, 1 .0) |  | 1 .0 (0.25, 2 .0) | 1 .0 (0 .0, 1 .0) |  |
| Minimum-Maximum | 0 .0-4 .0 | 0 .0-2 .0 |  | 0 .0-5 .0 | 0 .0-3 .0 |  |
| Unknown | 5 | 9 |  | 9 | 14 |  |
| Relaxation score |  |  | 0.3 |  |  | 0.4 |
| Median (IQR) | 3 .0 (0 .0, 3.25) | 3 .0 (0 .0, 3.25) |  | 1 .0 (1 .0, 2.50) | 3.50 (1.50, 4.75) |  |
| Minimum-Maximum | 0 .0-4 .0 | 0 .0-5 .0 |  | 0 .0-4 .0 | 0 .0-5 .0 |  |
| Unknown | 5 | 9 |  | 8 | 13 |  |
| ^1^Median (IQR) Range or Frequency (%) ^2^Wilcoxon signed rank test with continuity correction | | | | | | |
