# Supplementary material for: A randomised pilot study evaluating music therapy and virtual reality mindfulness sessions for reducing anxiety and stress in patients undergoing first-time elective cardiac surgery
Source: J Perioper Pract. 2025 Oct 4;36(1-2):59–67. doi: 10.1177/17504589251370291 (PMC12712224; doi:10.1177/17504589251370291)
Supplement: sj-docx-6-ppj-10.1177_17504589251370291 – Supplemental material for A randomised pilot study evaluating music therapy and virtual reality mindfulness sessions for reducing anxiety and stress in patients undergoing first-time elective cardiac surgery [file sj-docx-6-ppj-10.1177_17504589251370291.docx]

**Supplementary Table 4 – Cortisol levels before surgery**

|  | **Music** | | | **VR** | | |
| --- | --- | --- | --- | --- | --- | --- |
|  | **Pre-Music**  N = 17 | **Post-Music**  N = 17 | **p-value** | **Pre-VR**  N = 18 | **Post-VR**  N = 18 | **p-value** |
| **Cortisol** |  |  | 0.2^1^ |  |  | 0.11^1^ |
| Median (IQR) | 6.0 (4.8, 7.6) | 5.6 (4.1, 7.7) |  | 4.45 (3.05, 8.53) | 4.45 (2.73, 6.83) |  |
| Range | 2.0-27.6 | 2.2-16.9 |  | 1.80-13.60 | 1.30-16.70 |  |
| Change  Median (IQR) | -0.70(-2.30,0.70) | | -0.60(-1.10,0.15) | | | 0.7^2^ |
| ^1^Wilcoxon signed rank test with continuity correction (paired)  ^2^ Mann-Whitney U test | | | | | | |
| **Cortisol levels after surgery** | | | | | | |

|  | **Music** | | | **VR** | | |
| --- | --- | --- | --- | --- | --- | --- |
|  | **Pre-Music**  N = 11 | **Post-Music**  N = 11 | **p-value** | **Pre-VR**  N = 10 | **Post-VR**  N = 9 | **p-value** |
| **Cortisol** |  |  | 0.3^1^ |  |  | 0.3^1^ |
| Median (IQR) | 5 (2, 7) | 5 (3, 5) |  | 4.1 (2.8, 8.3) | 4.4 (4.0, 7.5) |  |
| Range | 1-27 | 1-21 |  | 0.9-19.4 | 0.9-16.9 |  |
| Change  Median (IQR) | -0.20(-1.80,0) | |  | -1.25(-2.58,0.08) | | 0.8^2^ |
| ^1^Wilcoxon signed rank test with continuity correction (paired)  ^2^ Mann-Whitney U test | | | | | | |
