# Supplementary material for: A randomised pilot study evaluating music therapy and virtual reality mindfulness sessions for reducing anxiety and stress in patients undergoing first-time elective cardiac surgery
Source: J Perioper Pract. 2025 Oct 4;36(1-2):59–67. doi: 10.1177/17504589251370291 (PMC12712224; doi:10.1177/17504589251370291)
Supplement: sj-docx-7-ppj-10.1177_17504589251370291 – Supplemental material for A randomised pilot study evaluating music therapy and virtual reality mindfulness sessions for reducing anxiety and stress in patients undergoing first-time elective cardiac surgery [file sj-docx-7-ppj-10.1177_17504589251370291.docx]

**Supplementary table 5- VR and MT Experience scores After Surgery**

| **Characteristic** | **Overall**, N = 36^1^ | **Music**, N = 17^1^ | **VR**, N = 19^1^ | **p-value**^2^ |
| --- | --- | --- | --- | --- |
| How did you felt before starting the headset session |  |  |  | 0.3 |
| Median (IQR) | 3.00 (2.25, 3.00) | 3.00 (2.00, 3.00) | 3.00 (3.00, 4.00) |  |
| Unknown | 14 | 6 | 8 |  |
| How stressed are you before starting the headset session |  |  |  | 0.13 |
| Median (IQR) | 1.00 (0.00, 2.00) | 1.00 (1.00, 2.00) | 0.00 (0.00, 1.00) |  |
| Unknown | 16 | 6 | 10 |  |
| How calm you are before using the headset session |  |  |  | 0.2 |
| Median (IQR) | 1.00 (1.00, 2.00) | 1.00 (1.00, 1.50) | 2.00 (1.00, 2.00) |  |
| Unknown | 16 | 6 | 10 |  |
| Using the headset session was a pleasurable experience |  |  |  | 0.11 |
| Median (IQR) | 4.00 (4.00, 4.00) | 4.00 (3.50, 4.00) | 4.00 (4.00, 4.00) |  |
| Unknown | 16 | 6 | 10 |  |
| After using the headset session, I felt relaxed |  |  |  | 0.2 |
| Median (IQR) | 4.00 (3.00, 4.00) | 4.00 (4.00, 4.00) | 4.00 (3.00, 4.00) |  |
| Unknown | 16 | 6 | 10 |  |
| After using the headset session, I felt less stressed compared to before attending the session |  |  |  | 0.9 |
| Median (IQR) | 4.00 (3.00, 4.00) | 4.00 (3.00, 4.00) | 4.00 (3.00, 4.00) |  |
| Unknown | 16 | 6 | 10 |  |
| After using the headset session, I felt calmer |  |  |  | 0.12 |
| Median (IQR) | 3.00 (3.00, 4.00) | 4.00 (3.00, 4.00) | 3.00 (3.00, 3.25) |  |
| Unknown | 18 | 7 | 11 |  |
| Using the headset enhanced my mood |  |  |  | 0.2 |
| Median (IQR) | 4.00 (3.00, 4.00) | 4.00 (3.50, 4.00) | 3.00 (3.00, 4.00) |  |
| Unknown | 16 | 6 | 10 |  |
| Using the headset session made me to think about doing more to prioritise self-care |  |  |  | 0.032 |
| Median (IQR) | 3.00 (3.00, 4.00) | 4.00 (3.00, 4.00) | 3.00 (2.00, 3.00) |  |
| Unknown | 16 | 6 | 10 |  |
| Using the headset made me to feel uncomfortable uneasy |  |  |  | 0.5 |
| Median (IQR) | 0.00 (0.00, 0.00) | 0.00 (0.00, 0.00) | 0.00 (0.00, 0.00) |  |
| Unknown | 15 | 6 | 9 |  |
| Using the headset made me to feel vomiting |  |  |  | >0.9 |
| Median (IQR) | 0.00 (0.00, 0.00) | 0.00 (0.00, 0.00) | 0.00 (0.00, 0.00) |  |
| Unknown | 14 | 6 | 8 |  |
| Using the headset made me to feel dizzy (lightheaded) |  |  |  | 0.4 |
| Median (IQR) | 0.000 (0.000, 0.000) | 0.000 (0.000, 0.000) | 0.000 (0.000, 0.000) |  |
| Unknown | 16 | 6 | 10 |  |
| Using the headset made me to feel nausea |  |  |  | 0.9 |
| Median (IQR) | 0.00 (0.00, 0.00) | 0.00 (0.00, 0.00) | 0.00 (0.00, 0.00) |  |
| Unknown | 15 | 6 | 9 |  |
| Using the headset made me to feel claustrophobia |  |  |  | >0.9 |
| Median (IQR) | 0.00 (0.00, 0.00) | 0.00 (0.00, 0.00) | 0.00 (0.00, 0.00) |  |
| Unknown | 17 | 7 | 10 |  |
| Using the headset made me to itch my facial skin |  |  |  | >0.9 |
| Median (IQR) | 0.00 (0.00, 0.00) | 0.00 (0.00, 0.00) | 0.00 (0.00, 0.00) |  |
| Unknown | 16 | 6 | 10 |  |
| Using the headset made me to feel headache |  |  |  | >0.9 |
| Median (IQR) | 0.00 (0.00, 0.00) | 0.00 (0.00, 0.00) | 0.00 (0.00, 0.00) |  |
| Unknown | 16 | 6 | 10 |  |
| Using the headset was fun and cool |  |  |  | >0.9 |
| Median (IQR) | 4.00 (3.00, 4.00) | 4.00 (3.25, 4.00) | 4.00 (3.00, 4.00) |  |
| Unknown | 17 | 7 | 10 |  |
| I would strongly recommend future patients to use headset before going to surgery |  |  |  | 0.7 |
| Median (IQR) | 4.00 (3.75, 4.00) | 4.00 (3.25, 4.00) | 4.00 (4.00, 4.00) |  |
| Unknown | 16 | 7 | 9 |  |
| Self-care |  |  |  | >0.9 |
| 1 hour a day | 1 (6.7%) | 0 (0%) | 1 (13%) |  |
| 1Hr per week | 1 (6.7%) | 0 (0%) | 1 (13%) |  |
| 2 hours | 1 (6.7%) | 0 (0%) | 1 (13%) |  |
| 3-4 hours | 1 (6.7%) | 0 (0%) | 1 (13%) |  |
| 4-5 times per week | 1 (6.7%) | 0 (0%) | 1 (13%) |  |
| 5 mins | 1 (6.7%) | 0 (0%) | 1 (13%) |  |
| Half a day | 3 (20%) | 2 (29%) | 1 (13%) |  |
| Never | 1 (6.7%) | 1 (14%) | 0 (0%) |  |
| Not much | 1 (6.7%) | 1 (14%) | 0 (0%) |  |
| Once a day | 1 (6.7%) | 1 (14%) | 0 (0%) |  |
| Once a week | 3 (20%) | 2 (29%) | 1 (13%) |  |
| Unknown | 21 | 10 | 11 |  |
| ^1^Median (IQR) or Frequency (%) | | | | |
| ^2^Wilcoxon rank sum test; Fisher's exact test | | | | |

| **Characteristic** | **N = 9** |
| --- | --- |
| Absorption |  |
| Median (IQR) | 100 (90, 100) |
| Immersion |  |
| Median (IQR) | 90 (80, 90) |

**Absorption and immersion after surgery**
